# Supplementary material for: Somatic hypermutation patterns are shaped by both motif position and sequence grammar
Source: EMBO J. 2025 Dec 11;45(3):879–900. doi: 10.1038/s44318-025-00640-9 (PMC12864871; doi:10.1038/s44318-025-00640-9)
Supplement: Supplementary file 4 — Expanded View Figures [file 44318_2025_640_MOESM4_ESM.pdf]

Expanded View Figures

Figure EV1. Analysis of positional differences in mutation frequency of WRCH motifs.

(**A**) Mutation frequency of the central C within all cytosine-centered 15-mers (left panel) or of the central C within WRCH-centered 15-mers (right panel). The boxes span the interquartile range with the median represented by the horizontal line. The dot size corresponds to the number of unique 15-mers represented by that dot. The numbers of unique 15-mers (*n*) are indicated in brackets. Box plots were plotted with ggplot2 (https://doi.org/10.1007/978-3-319-24277-4). The lower and upper hinges correspond to the first and third quartiles (the 25th and 75th percentiles). The upper whisker extends from the hinge to the largest value no further than 1.5* IQR from the hinge (where IQR is the interquartile range, or distance between the first and third quartiles). The lower whisker extends from the hinge to the smallest value at most 1.5* IQR of the hinge. Data beyond the end of the whiskers are outlier points and are plotted individually. (**B**) Box plots, plotted as above, as above showing the mutation frequency of the central C within the twelve WRCH motif groups that are further categorized by their location within the indicated V sub-regions (FWR1, CDR1, FWR2, CDR2, and FWR3). The values in brackets are the number of unique 15-mers in that group.

 © The Author(s)

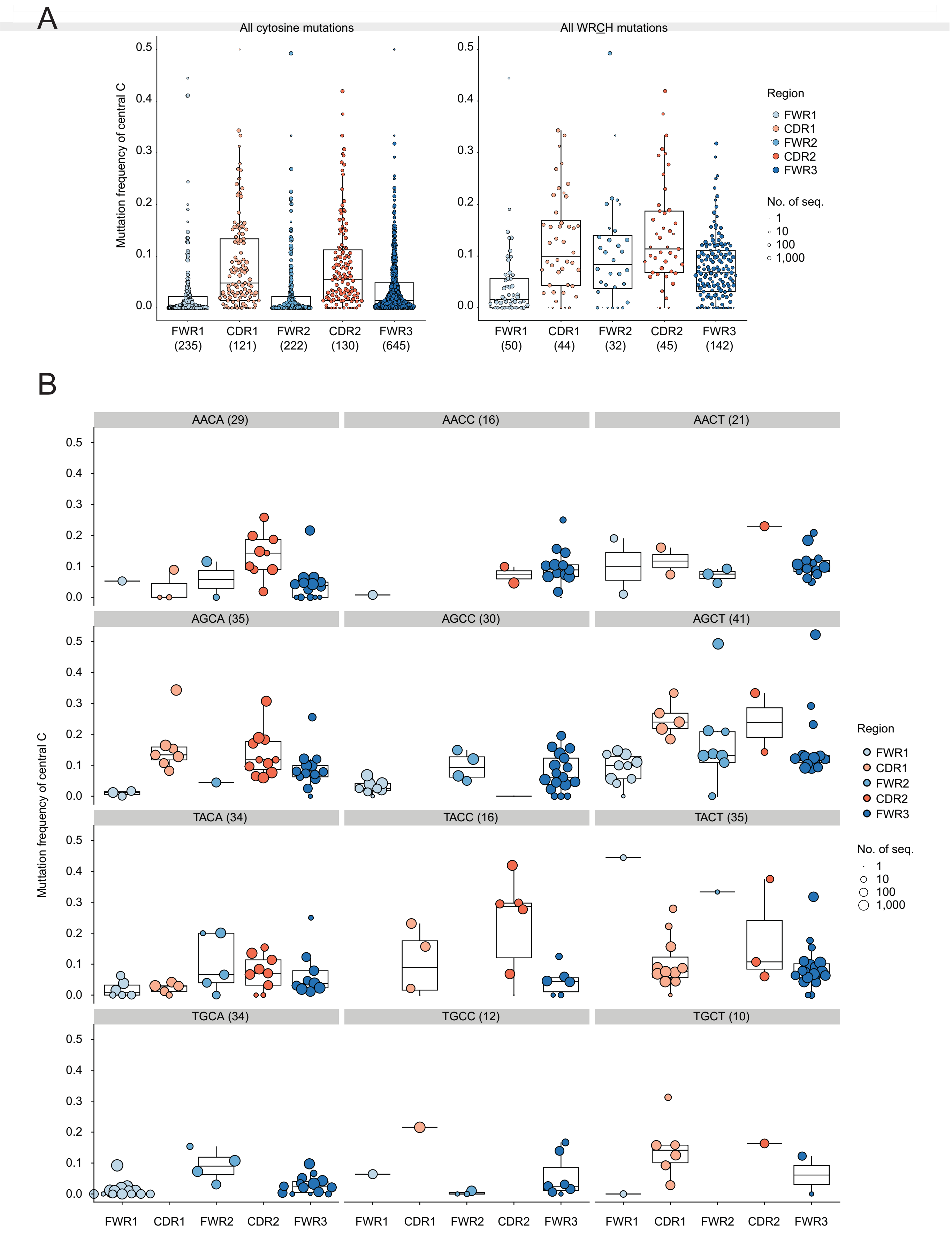
A
All cytosine mutations
All WRCH mutations
Mutation frequency of central C
0.5
0.4
0.3
0.2
0.1
0.0
FWR1 (235)
CDR1 (121)
FWR2 (222)
CDR2 (130)
FWR3 (645)
FWR1 (50)
CDR1 (44)
FWR2 (32)
CDR2 (45)
FWR3 (142)
Region
FWR1
CDR1
FWR2
CDR2
FWR3
No. of seq.
1
10
100
1,000
B
AACA (29)
AACC (16)
AACT (21)
AGCA (35)
AGCC (30)
AGCT (41)
TACA (34)
TACC (16)
TACT (35)
TGCA (34)
TGCC (12)
TGCT (10)
Mutation frequency of central C
FWR1
CDR1
FWR2
CDR2
FWR3

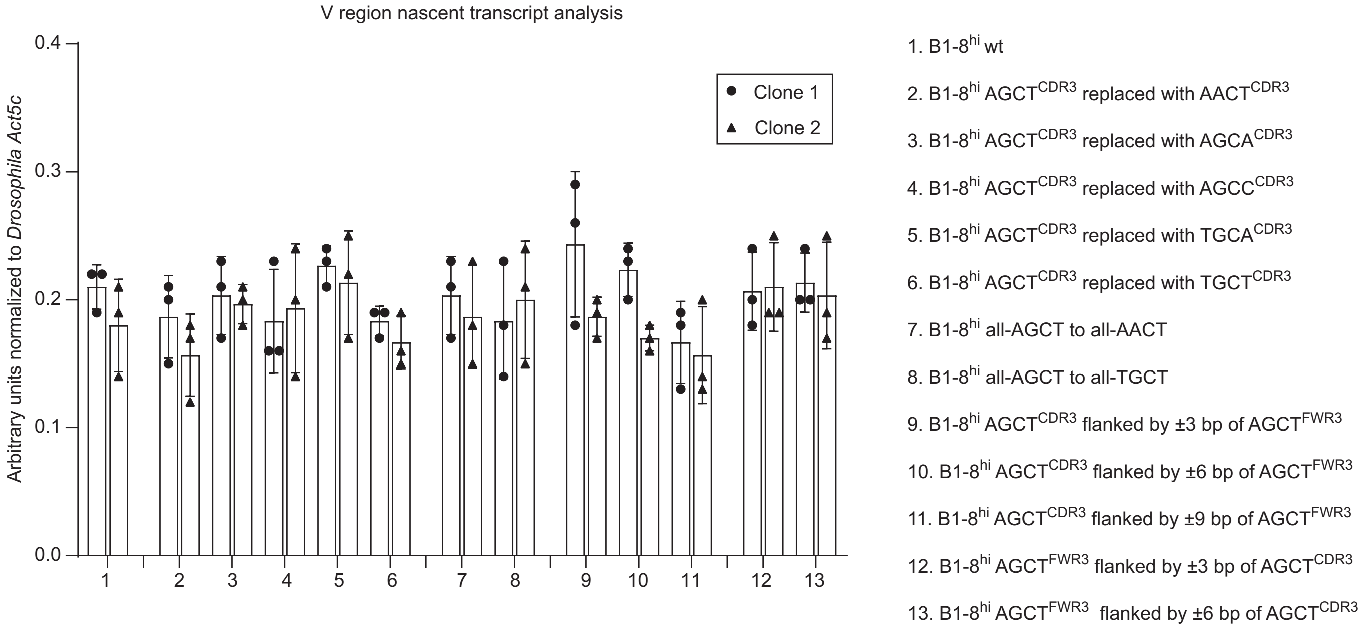

Figure EV2. Nascent transcription analysis of the V region from all the cell lines used in this study.

Three replicates from each clone were analyzed ($n = 3$). The error bars show the standard deviation. No significant changes were observed between the Ramos B1-8^{hi} wt cells (column 1) and any other cell line based on the unpaired, two-tailed Student's *t*-test.

 © The Author(s)
